# Obstructive sleep apnea is associated with impaired renal function in patients with diabetic kidney disease

**DOI:** 10.1038/s41598-021-85023-w

**Published:** 2021-03-11

**Authors:** Ester Zamarrón, Ana Jaureguizar, Aldara García-Sánchez, Trinidad Díaz-Cambriles, Alberto Alonso-Fernández, Vanesa Lores, Olga Mediano, Paula Rodríguez-Rodríguez, Sheila Cabello-Pelegrín, Enrique Morales-Ruíz, María T. Ramírez-Prieto, María Isabel Valiente-Díaz, Teresa Gómez-García, Francisco García-Río, Spanish Sleep Network, Beatriz Arias-Melgar, Antonia Barceló-Bennasar, Beatriz Barquiel, Ana Candel-Pizarro, Raquel Casitas, Olga Costero, Mónica De-La-Peña-Bravo, Ana María Díaz-Rubio, Raúl Galera, María Paloma Giménez-Carrero, Héctor Lozano-Alcocer, Alberto Mangas, Elizabet Martínez-Cerón, José Antonio Peña-Zarza, Rocío Rodríguez-Pérez, Sofía Romero-Peralta, Laura Silgado, Fernanda Troncoso-Acevedo

**Affiliations:** 1 Servicio de Neumología, Hospital Universitario La Paz-IdiPAZ, Paseo de La Castellana 261, 28046 Madrid, Spain; 2 Servicio de Neumología, Hospital Universitario Ramón y Cajal, Madrid, Spain; 3 Servicio de Neumología, Hospital Universitario, 12 de Octubre, Madrid, Spain; 4 Centro de Investigación Biomédica en Red en Enfermedades Respiratorias (CIBERES), Madrid, Spain; 5 Servicio de Neumología, Hospital Universitario Son Espases, Palma de Mallorca, Spain; 6 Institut d’Investigació Sanitària Illes Balears (IdISBa), Palma de Mallorca, Spain; 7 Servicio de Neumología, Hospital Universitario Infanta Sofía, San Sebastián de Los Reyes, Madrid, Spain; 8 Servicio de Neumología, Hospital Universitario de Guadalajara, Guadalajara, Spain; 9 Universidad de Alcalá, Madrid, Spain; 10 Servicio de Neumología, Fundación Jiménez Díaz, Madrid, Spain; 11 Servicio de Nefrología, Hospital Universitario Son Espases, Palma de Mallorca, Spain; 12 Servicio de Nefrología, Hospital Universitario, 12 de Octubre, Madrid, Spain; 13 Departamento de Medicina, Facultad de Medicina, Universidad Autónoma de Madrid, Madrid, Spain; 14 Servicio de Endocrinología, Hospital Universitario La Paz, Madrid, Spain; 15 Servicio de Nefrología, Hospital Universitario La Paz, Madrid, Spain

**Keywords:** Diseases, Endocrinology, Nephrology

## Abstract

Obstructive sleep apnea (OSA) is a recognized risk factor for the development of diabetic kidney disease (DKD). Our objectives were to compare the urinary albumin–creatinine ratio (UACR) and estimated glomerular filtration rate (eGFR) of patients with DKD according to OSA severity, and to evaluate the contribution of sleep parameters to their renal function. In a multicenter, observational, cross-sectional study, 214 patients with DKD were recruited. After a sleep study, UACR and eGFR were measured, as well as serum creatinine, fasting glucose, glycated hemoglobin, insulin resistance, lipid profile and C-reactive protein. UACR was higher in severe OSA patients (920 ± 1053 mg/g) than in moderate (195 ± 232 mg/g, p < 0.001) or mild OSA/non-OSA subjects (119 ± 186 mg/g, p < 0.001). At the same time, eGFR showed an OSA severity-dependent reduction (48 ± 23 vs. 59 ± 21 vs. 73 ± 19 ml/min per 1.73 m^2^, respectively; p < 0.001). Apnea–hypopnea index (AHI and desaturation index (ODI) were identified as independent predictors for UACR and eGFR, respectively. Therefore, in patients with DKD under optimized treatment, severe OSA is associated with a higher UACR and a lower eGFR, reflecting an additional contribution to the impairment of their renal function, although no causality can be inferred.

## Introduction

Obstructive sleep apnea (OSA) and type 2 diabetes are common diseases. It has been estimated that 936 million adults around the world have mild to severe OSA^1^, while type 2 diabetes affects 425 million people^2^. Progressive evidence shows that OSA is associated with impaired glucose tolerance, insulin resistance and increased risk of developing type 2 diabetes^3–5^. Furthermore, OSA could aggravate the evolution of diabetes, since it has an adverse effect on glycemic control^6,7^.

Among the main consequences of the progression of diabetes, diabetic kidney disease (DKD) deserves special consideration. This frequent diabetic microvascular complication is the main type of chronic kidney disease and the leading cause of renal failure and dialysis worldwide^8,9^. Although there are some conflicting results^10–12^, studies conducted in type 2 diabetes patients (mainly cross-sectional) have shown that mild to severe OSA (defined by an apnea–hypopnea index (AHI) ≥ 5/h) is associated with a higher prevalence of DKD, while also reporting a relationship between AHI and albuminuria^13–16^. The longitudinal analysis of a cohort of type 2 diabetes patients has even identified that the presence of OSA is associated with greater deterioration of the estimated glomerular filtration rate (eGFR)^17^. Thus, there is moderate evidence that OSA may be a risk factor for the development of DKD^18^.

Although treatment of DKD has slowed the rate of progression to end-stage renal disease, treatment costs are very high and do not prevent a high death rate^19^. Therefore, there is a need for new approaches that would allow the identification of additional risk factors to progression of DKD. Until now, OSA has been shown to be a risk factor for cardiovascular events and mortality in dialysis patients^20^, and several factors have linked OSA with the development and progression of chronic kidney disease, including diabetes mellitus, hypertension, arterial stiffness, proteinuria and obesity^21^. However, to our knowledge, information about the effect of OSA on the control of patients with an already established DKD is scarce or non-existent.

Therefore, our objective has been to compare the kidney function impairment, assessed in terms of urinary albumin–creatinine ratio (UACR) and eGFR, of patients with a previous diagnosis of DKD adequately treated according to conventional guidelines, based on the severity of associated OSA. We also intend to evaluate the contribution of sleep parameters to UACR and eGFR in these patients.

## Results

Table 1 shows the main characteristics of the 214 patients selected with a previous diagnosis of DKD. There was a predominance of men in the sixth-seventh decade of life, with a notable cumulative burden of smoking and comorbidities. 87.9% of the patients had a clinical diagnosis of dyslipidemia, 44.4% diabetic retinopathy, 26.6% ischemic heart disease, 15.0% autonomic neuropathy and 7.9% chronic heart failure. On average, the diagnosis of diabetes had been established more than 15 years prior to the study and that of DKD at least 4 years prior. In addition to oral antidiabetic drugs and insulin, a high percentage of patients currently used angiotensin II receptor blockers (ARB) and/or angiotensin-converting enzyme inhibitors.

**Table 1:** Comparison of general characteristics between patients with diabetic kidney disease according to obstructive sleep apnea.

	Total	Non OSA or mild OSA	Moderate OSA	Severe OSA	*p* value
N	214	38	65	111	–
Sex					0.576
Females, n (%)	49 (22.9)	10 (26.3)	12 (18.5)	27 (24.3)	
Males, n (%)	165 (77.1)	28 (73.7)	53 (81.5)	84 (75.7)	
Age (years)	67 ± 10	67 ± 9	65 ± 10	69 ± 9^§^	0.011
BMI (kg/m^2^)	31.6 ± 5.0	28.9 ± 3.8	31.0 ± 4.2	32.9 ± 5.5^†‡^	< 0.001
FMI (kg/m^2^)	11.6 ± 5.0	11.8 ± 4.4	10.9 ± 5.1	12.0 ± 5.1	0.439
Neck circumference (cm)	43 ± 4	42 ± 5	42 ± 4	43 ± 4	0.053
Waist–hip ratio	1.02 ± 0.09	1.01 ± 0.09	1.00 ± 0.08	0.03 ± 0.09	0.075
Smoking status					0.185
Current smoker, n (%)	33 (15.6)	5 (13.2)	15 (23.8)	13 (11.7)	
Former smoker, n (%)	111 (52.4)	21 (55.3)	33 (52.4)	57 (51.4)	
Never smoker, n (%)	68 (32.1)	12 (31.6)	15 (23.8)	41 (36.9)	
Packs × year	46 ± 32	36 ± 32	46 ± 35	49 ± 29	0.241
Duration of diabetes (years)	14 (9–21)	14 (7–20)	15 (10–21)	14 (9–21)	0.642
Duration of diabetic kidney disease (years)	3 (1–6)	3 (1–5)	3 (1–5)	2 (1–6)	0.659
Heart rate (/min)	77 ± 12	79 ± 12	78 ± 13	76 ± 12	0.458
SBP (mmHg)	141 ± 19	145 ± 19	141 ± 18	141 ± 20	0.537
DBP (mmHg)	77 ± 12	77 ± 14	76 ± 12	78 ± 12	0.780
Current treatment					
Oral antidiabetic drugs, n (%)	179 (83.6)	33 (86.8)	51 (78.5)	95 (85.6)	0.393
Insulin, n (%)	136 (63.6)	20 (52.6)	44 (67.7)	72 (64.9)	0.284
ACEi, n (%)	77 (36.0)	15 (39.5)	26 (40.0)	36 (32.4)	0.532
ARBs, n (%)	122 (57.0)	23 (60.5)	32 (49.2)	67 (60.4)	0.316
MRAs, n (%)	18 (8.4)	5 (13.2)	7 (10.8)	6 (5.4)	0.237
Age-adjusted Charlson index	7.1 ± 2.5	7.0 ± 2.2	6.6 ± 2.8	7.3 ± 2.5	0.196
Physical activity level					0.319
Low, n (%)	29 (18.1)	2 (8.7)	11 (21.6)	16 (18.6)	
Moderate, n (%)	103 (64.4)	15 (65.2)	29 (56.9)	59 (68.6)	
High, n (%)	28 (17.5)	6 (26.1)	11 (21.6)	11 (12.8)	

Values are number (percentage), median (interquartile range [IQR]) or mean ± SD, according their characteristics and distribution. Comparisons were performed by chi-squared test, Kruskal–Wallis test or ANOVA with post-hoc comparisons by Bonferroni test: ^†^*p* < 0.001 vs. non-OSA or mild OSA; ^‡^*p* < 0.05, ^§^*p* < 0.01 vs. moderate OSA.

*OSA* obstructive sleep apnea, *BMI* body mass index, *FMI* fat mass index, *SBP* systolic blood pressure, *DBP* diastolic blood pressure, *ACEi* angiotensin-converting enzyme inhibitors, *ARB* angiotensin II receptor blockers, *MRA* aldosterone receptor antagonists.

### Sleep characteristics of patients with diabetic kidney disease

According to Epworth sleepiness scale, daytime hypersomnia reported in our study was mild in 116 patients (59.5%), moderate in 54 (27.7%) and severe in 4 (2.1%), with a overall score of 7 (4–9). The AHI was 30.0 (17.9–46.2)/h, with a desaturation index of 31.3 (19.3–47.9)/h and a predominance of obstructive events (94 ± 11%). During the 30.3 (10.9–53.8)% of the recording time, the patients had oxygen saturation less than 90%, with a mean and a low nocturnal oxygen saturation of 91 (89–92) and 77 (69–82)%, respectively. According to conventional criteria, OSA was identified in 212 patients, which was mild in 36 cases (16.8%), moderate in 65 (30.4%) and severe in 111 (51.9%).

The comparison of patients with DKD without OSA or with mild OSA versus those with moderate OSA or severe OSA only showed a slight difference in age and BMI, which were both greater in severe OSA patients. There were no differences between the three groups in other anthropometric characteristics, smoking, time of evolution of diabetes or DKD, blood pressure, comorbidity, level of daily physical activity or current treatment (Table 1).

### Kidney involvement is increased in DKD patients with severe OSA

**Figure 1:**
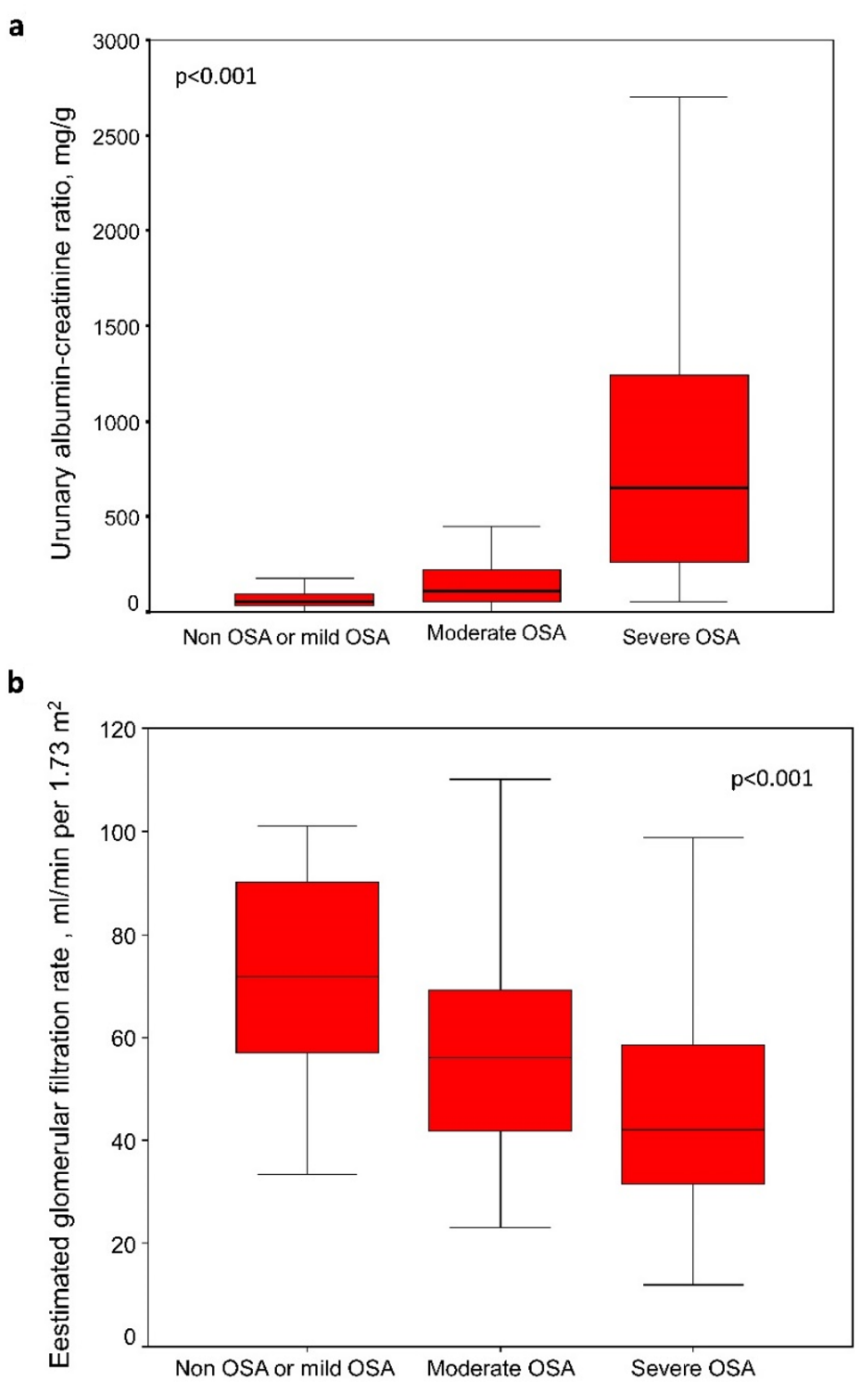
Box-and-whisker plots depicting the distribution of (**a**) urinary albumin–creatinine ratio and (**b**) estimated glomerular filtration rate of patients with diabetic kidney disease according to OSA severity. The dark line in the middle of the boxes represents the median and the length of the box reflects the interquartile range (IQR). The T-bars represent maximum and minimum values. Comparisons performed using SPSS 26.0 software (SPSS Inc., Chicago, IL, USA).

In addition to the evident differences in daytime sleepiness and sleep parameters according to the presence and severity of OSA (Table 2), differences in albuminuria and glomerular filtration were also identified. Patients with DKD and severe OSA had a higher UACR than patients with moderate OSA and those without OSA or with mild OSA (Fig. 1a). Similarly, an OSA severity-dependent reduction in eGFR has been also found (Fig. 1b), as well as increased serum creatinine levels in patients with severe OSA. In contrast, there were no differences between the three groups in fasting glucose, glycated hemoglobin, insulin resistance or sensitivity, lipid profile, or serum level of high sensitivity C-reactive protein.

**Table 2:** Comparison of sleep characteristic and biochemical parameters between the OSA subgroups.

	Non OSA or mild OSA	Moderate OSA	Severe OSA	*p* value
Epworth sleepiness scale	6 (4–7)	6 (3–8)	8 (5–11)	0.001
AHI (/h)	11.8 (7.6–13.6)	21.7 (17.2–25.5)	46.9 (37.6–59.9)	< 0.001
Desaturation index (/h)	12.0 (8.0–14.1)	23.1 (18.4–26.9)	47.7 (37.6–59.7)	< 0.001
tSaO_2_ < 90% (%)	9 (2–43)	19 (6–49)	37 (14–60)	0.001
Mean nocturnal SaO_2_ (%)	92 (90–93)	91 (90–92)	90 (88–92)	0.019
Low nocturnal SaO_2_ (%)	83 (79–86)	80 (74–83)	74 (65–79)	< 0.001
Apnea index (/h)	1.8 (0.6–3.0)	5.5 (2.9–9.7)	17.3 (10.7–30.9)	< 0.001
Hypopnea index (/h)	9.2 (5.6–11.1)	15.6 (10.8–19.4)	24.8 (17.7–32.6)	< 0.001
Obstructive apnea index (/h)	1.5 (0.3–3.0)	4.6 (2.3–9.0)	12.8 (6.8–23.8)	< 0.001
Central apnea index (/h)	0 (0–0.2)	0.5 (0–1.0)	0.8 (0–4.5)	< 0.001
Urinary albumin–creatinine ratio (mg/g)	57.6 (35.1–96.4)	110.3 (53.0–217.9)	651.9 (259.6–1242.8)	< 0.001
eGFR (ml/min per 1.73 m^2^)	71.9 (57.0–90.0)	56.0 (41.8–69.3)	42.0 (31.6–58.5)	< 0.001
Serum creatinine (mg/dl)	1.4 ± 0.5	1.3 ± 0.5	1.6 ± 0.8^**#**^	0.007
Fasting glucose (mg/dl)	137 (113–179)	144 (113–185)	150 (123–180)	0.653
HbA1c (%)	7.4 ± 1.5	7.8 ± 1.8	7.6 ± 1.4	0.470
HbA1c (mmol/mol)	57.1 ± 16.4	61.3 ± 19.9	59.9 ± 15.2	0.470
HOMA-IR index^a^	4.59 (1.73–7.83)	3.30 (2.42–6.20)	5.36 (2.92–8.54)	0.405
QUICKI index^a^	0.31 (0.29–0.35)	0.32 (0.29–0.33)	0.30 (0.28–0.33)	0.405
hsCRP (mg/l)	0.80 (0.20–3.30)	1.80 (0.40–4.15)	2.75 (0.70–6.10)	0.103
Total cholesterol (mg/dl)	152 ± 33	157 ± 41	149 ± 36	0.386
HDLc (mg/dl)	40 ± 10	42 ± 12	40 ± 13	0.461
LDLc (mg/dl)	85 ± 27	83 ± 39	80 ± 30	0.701
Triglycerides (mg/dl)	152 ± 76	184 ± 114	153 ± 66	0.051

Values are median (interquartile range [IQR]) or mean ± SD, according their distribution. Comparisons were performed by Kruskal–Wallis test or ANOVA with post-hoc comparisons by Bonferroni test: ^#^*p* < 0.01 vs. moderate OSA.

*AHI* apnea–hypopnea index, *tSaO*_*2*_ percentage of recording time with oxygen saturation < 90%, *SaO*_*2*_ oxygen saturation, *eGFR* estimated glomerular filtration rate, *HbA1c* glycated hemoglobin, *HOMA-IR* Homeostasis Model Assessment-Insulin Resistance (HOMA-IR), *QUICKI* Qualitative Insulin Sensitivity Check Index, *hsCRP* high-sensitivity C reactive protein, *HDL* high-density lipoprotein cholesterol, *LDL-c* low-density lipoprotein cholesterol.

^a^Only in noninsulin user patients (n = 78).

### Determinants of kidney function in patients with diabetic kidney disease

**Figure 2:**
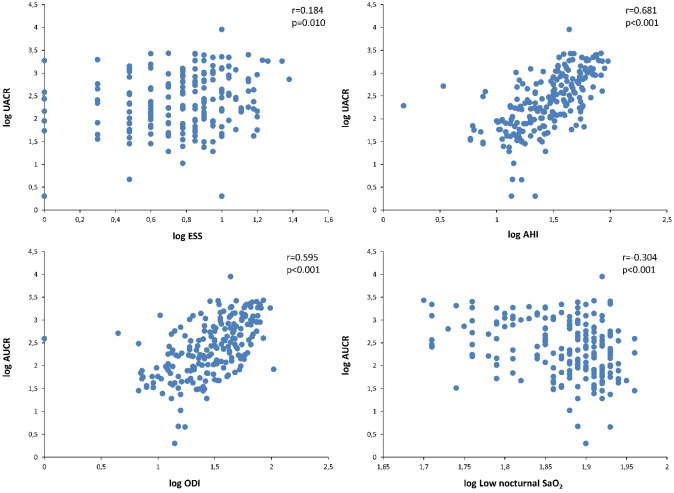
Relationship between the sleep characteristics and the urinary albumin–creatinine ratio (UACR) in patients with diabetic kidney disease. Data are log transformed. *ESS* Epworth sleepiness scale, *AHI* apnea–hypopnea index, *ODI* desaturation index, *SaO*_*2*_ oxygen saturation.

**Figure 3:**
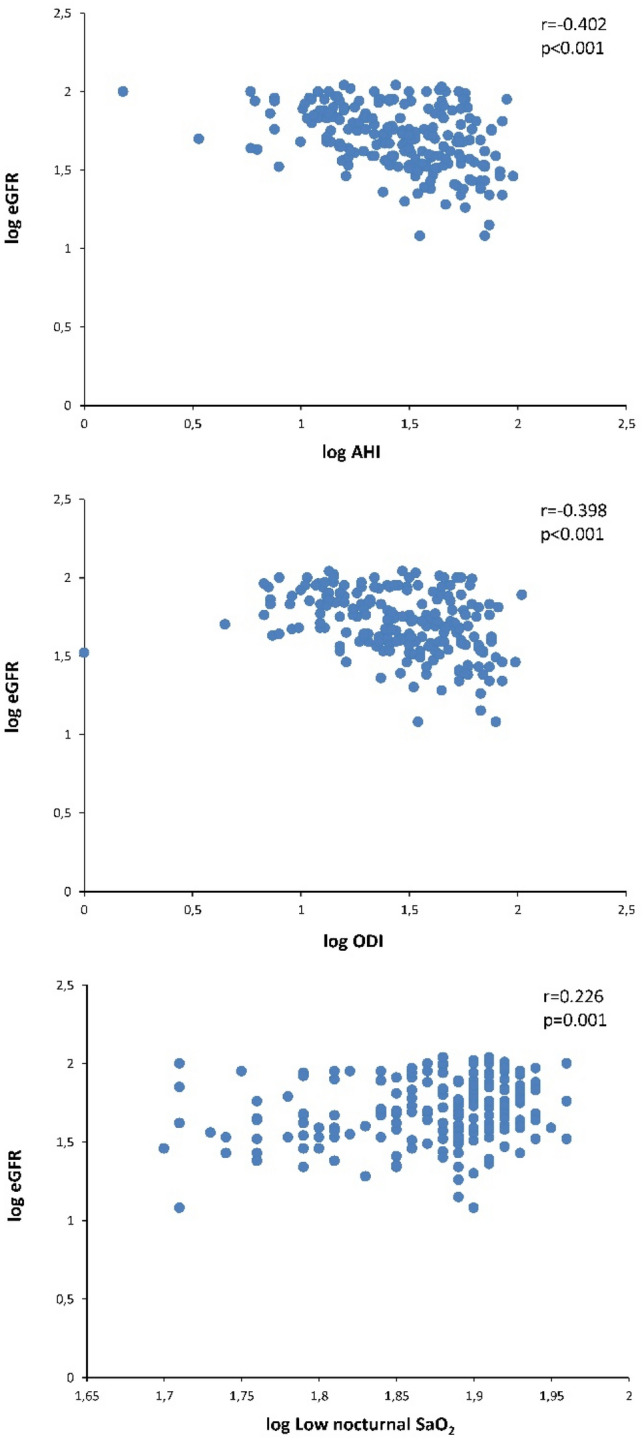
Relationship between the sleep characteristics and the estimated glomerular filtration rate (eGFR) in patients with diabetic kidney disease. Data are log transformed. *AHI* apnea–hypopnea index, *ODI* desaturation index, *SaO*_*2*_ oxygen saturation.

Overall, the UACR in patients with DKD was directly related to the Epworth sleepiness scale, AHI and the desaturation index, while it maintained an inversely proportional relationship with low nocturnal SaO_2_ (Table 3 and Fig. 2). In turn, the eGFR was inversely related to the AHI and desaturation index and directly correlated with low nocturnal SaO_2_ (Fig. 3). In contrast, fasting glucose and HbA1c level were not related to any sleep parameter, while insulin resistance or sensitivity indices were only related to the oximetry variables. HOMA-IR was related to tSaO_2_ < 90% (r = 0.325, *p* = 0.009), mean nocturnal SaO_2_ (r = − 0.232, *p* = 0.043) and low nocturnal SaO_2_ (r = − 0.240, *p* = 0.038), while the QUICKI index did so with tSaO_2_ < 90% (r = − 0.325, *p* = 0.009) and lowest nocturnal SaO_2_ (r = 0.240, *p* = 0.037). In turn, serum levels of hsCRP were related to AHI (r = 0.188, p = 0.016), mean nocturnal SaO_2_ (r = − 0.182, *p* = 0.020) and low nocturnal SaO_2_ (r = − 0.204, p = 0.009).

**Table 3:** Relationship between sleep characteristics and renal function parameters in patients with diabetic kidney disease.

Parameter	log UACR			log eGFR		
	r	95% CI	*p* value	r	95% CI	*p* value
log ESS	0.184	0.045–0.316	0.010	–	–	–
log AHI	0.681	0.602–0.747	< 0.001	–0.402	− 0.509 to − 0.283	< 0.001
log ODI	0.595	0.499–0.676	< 0.001	− 0.398	− 0.507 to − 0.277	< 0.001
log lowest SaO_2_, %	− 0.304	− 0.424 to − 0.174	< 0.001	0.226	0.091–0.352	0.001

Variables are log transformed and data recorded are Spearman’s correlation coefficient, 95% confidence interval of the correlation and p value.

*UACR* urinary albumin–creatinine ratio, *eGFR* estimated glomerular filtration rate, *ESS* Epworth sleepiness scale, *AHI* apnea–hypopnea index, *ODI* desaturation index, *SaO*_*2*_ oxygen saturation.

Finally, the multiple linear regression model adjusted for age, sex, BMI, fat mass index, duration of diabetes, smoking status, systolic and diastolic blood pressures, HbA1c, cholesterol, HDLc, LDLc, triglycerides, and treatment with insulin, ACEi, ARB or MRA drugs only retained AHI as an independent predictor of the UACR (standardized B coefficient = 0.460, p < 0.001). With regard to eGFR, the multiple linear regression model adjusted for the same variables only retained the desaturation index as an independent determinant (standardized B coefficient =  − 0.324, p < 0.001) (Table 4).

**Table 4:** Independent predictors of urinary albumin–creatinine ratio and estimated glomerular filtration rate in the multivariate linear regression analyses.

	Non-standardized coefficients			Standardized coefficients	r^2^	*p* value
	B	S.E	95% CI for B	B		
**Urinary albumin–creatinine ratio (mg/dl)**^**a**^						
AHI (/h)	23.3	3.8	15.9–30.7	0.460	0.212	< 0.001
Constant	− 237.6	150.8	− 535.8 to 60.5	–	–	0.117
**Estimated glomerular filtration rate (eGFR) (ml/min per 1.73 m**^**2**^)^**b**^						
ODI (/h)	− 0.41	0.10	− 0.61 to − 0.21	− 0.324	0.105	< 0.001
Constant	70.75	4.05	62.75 to 78.74	–	–	< 0.001

*AHI* apnea–hypopnea index, *ODI* desaturation index, *BMI* body mass index, *FMI* fat mass index, *SBP* systolic blood pressure, *DBP* diastolic blood pressure, *HbA1c* glycated hemoglobin, *HDLc* high-density lipoprotein cholesterol, *LDLc* low-density lipoprotein cholesterol, *eGFR* estimated glomerular filtration rate, *ACEi* angiotensin-converting enzyme inhibitors, *ARB* angiotensin II receptor blockers, *MRA* aldosterone receptor antagonists, *tSaO*_*2*_* < 90%* percentage of recording time with oxygen saturation < 90%, *SaO*_*2*_ oxygen saturation.

^a^Adjusted for age, sex, BMI, FMI, duration of diabetes, smoking habit, SBP, DBP, HbA1c, cholesterol, HDLc, LDLc, triglycerides, eGFR, treatment with insulin, ACEi, ARB or MRA drugs, desaturation index, tSaO_2_ < 90%, mean nocturnal SaO_2_ and lowest nocturnal SaO_2_.

^b^Adjusted for age, sex, BMI, FMI, duration of diabetes, smoking habit, SBP, DBP, HbA1c, cholesterol, HDLc, LDLc, triglycerides, treatment with insulin, ACEi, ARB or MRA drugs, AHI, tSaO_2_ < 90%, mean nocturnal SaO_2_ and lowest nocturnal SaO_2_.

## Discussion

The main results of our study are that patients with previously established diabetic kidney disease treated according to conventional clinical practice who present with severe OSA have a greater severity of kidney involvement (in terms of a higher urinary albumin–creatinine ratio and a lower estimated glomerular filtration rate) than non-apneic patients or those with mild OSA. Furthermore, we have identified that, in this group of patients, AHI is an independent determinant of UACR, while desaturation index is an independent determinant of eGFR.

One prominent finding is the high prevalence of OSA in our patients with DKD, but with little impact on sleep-related symptoms since the average score of the Epworth sleepiness scale was 7, and only 29.8% reported moderate to severe sleepiness, as usually happens in studies performed in patients not directly referred to a sleep unit^22^. These results could relatively agree with those obtained by Tahrani et al.^17^, who identified a prevalence of OSA of 79% among the 90 patients in their study who had DKD, despite having a lower prevalence of males, the patients being a decade younger and having a shorter diabetes evolution time. Interestingly, this study also found a notable discrepancy in the prevalence of OSA according to the ethnic group of patients with DKD, ranging from 89% in white Europeans to 67% in Southern Asians^17^. Similarly, in 880 Chinese type 2 diabetes patients hospitalized for poor glycemic control, microvascular complications, cerebrovascular complications, cardiovascular complications or infection, the prevalence of OSA was 9% in the 11% who had a previous history of DKD^14^. In addition to the ethnic differences compared to our patients, this study also recruited a higher percentage of women who were younger, had a lower BMI and had a shorter duration of diabetes. In any case, it is also necessary to highlight that our study may slightly overestimate the prevalence of OSA in patients with DKD because we have selected overweight or obese patients, in whom this disorder is more prevalent.

The selection of UACR and eGFR as indicators for the severity of DKD was due to their easy determination and universal access, in addition to being proven predictors of renal prognosis and not so much of cardiovascular prognosis, which seems more dependent on glycemic control and, therefore, appears to be less specific for renal involvement in diabetes^23^. In fact, eGFR and albuminuria have been shown to be independent predictors of renal outcomes in patients with DKD, whereas cardiovascular end-points are more dependent on age, HbA1c or phosphorus level^24^. The relevance of UACR and eGFR is confirmed in classic studies that identify them, together with pathological grade, as prognostic factors for the progression of DKD to end-stage renal disease^25^.

Although to our knowledge there is no exclusive information on patients with DKD, the identification of differences in UACR and eGFR based on the severity of OSA, stratified by AHI, coincides with previous studies in which this parameter has been identified as a risk factor for the development of albuminuria in patients with type 2 diabetes^11,13–15,17,26^. Similarly, other studies have also described that the desaturation index is a risk factor for the development of DKD^27,28^. However, our results are in contrast with the finding of Leong et al.^13^, who identified that the tSaO_2_ < 90% was related to the eGFR of patients with type 2 diabetes with or without DKD. When the former are specifically analyzed, as has been our case, the tSaO_2_ < 90% does not reach any correlation with the UACR or the eGFR.

In general, we consider that our findings have two clinical-pathogenic implications in the effect of OSA on DKD. First, they highlight that the impact is limited to patients with severe OSA and, to a lesser extent, moderate OSA. This finding provides a dimension of the problem that is more consistent with the prevalence of both entities and with the identification of risk groups. It also coincides in the need to identify AHI cut-off points higher than 5 to select patients with higher cardiovascular risk^29,30^. Secondly, the influence of AHI and the oxygen desaturation index on renal function variables instead of tSaO_2_ < 90% or median nocturnal SaO_2_ indicates that the impact of OSA is due to sleep fragmentation and/or hypoxia-reoxygenation episodes and not so much the maintenance of sustained hypoxemia during the night.

Another outstanding finding of our study is the verification that in the UACR or eGFR prediction models, the AHI or the desaturation index displace classic risk factors for a poor renal prognosis in patients with DKD, such as age, BMI, duration of diabetes or DKD, systolic blood pressure (SBP), HbA1c or presence of DKD^12^. Of all of them, the absence of a relationship between UACR and eGFR with HbA1c or SBP is particularly relevant, which are clearly altered parameters in patients with OSA. This poses the attractive hypothesis that OSA could worsen albuminuria or eGFR, regardless of poor glycemic control and high blood pressure, through the involvement of other pathogenic mechanisms.

Although our study does not intend to evaluate pathogenic pathways, in a purely speculative way it could be stated that several OSA-related pathophysiologic changes might influence glomerular endothelial function and urinary albumin excretion in patients with DKD through various pathways. First, sleep fragmentation activates the sympathetic nervous system and the renin–angiotensin–aldosterone system^31,32^. In addition to promoting the development of hypertension^33^, which seems not to be related to the renal function of our patients, this can also lead to tubulointerstitial injury^32,34^ and renal fibrosis^35^, causing proteinuria and decreased eGFR^31^. Moreover, sympathetic activation may also increase angiotensin II, leading to efferent arteriolar vasoconstriction and hyperfiltration, which is a key pathway for the progression of chronic kidney disease^36^. Second, OSA with concurrent type 2 diabetes is associated with increased oxidative and nitrosative stress and activation of the inflammatory pathways, impairing microvascular and endothelial regulation^32^. These induce damage in vulnerable endothelial and mesangial cells and peripheral nerves, which may promote the kidney damage^37,38^. Moreover, OSA-related reactive oxygen species and systemic inflammation contribute to atherosclerosis^31^ and may also contribute to chronic kidney disease progression^32^. And finally, OSA could induce intermittent intra-renal hemodynamic changes. Moreover, recurrent sympathetic overactivity due to sleep fragmentation also can cause intermittent intra-renal hemodynamic changes that induce ischemia and reperfusion injury, promoting the intrinsic renal damage^39^. In fact, in a case report on OSA that showed secondary focal glomerulosclerosis, proteinuria improved completely after treatment with bi-level positive airway pressure^40^. Moreover, patients with untreated OSA have a lower renal plasma flow and a higher filtration fraction compared to treated patients^41^.

Our study has several strengths. It is a multicenter study that specifically evaluates patients who already have established DKD, using proven indicators for kidney damage. In addition, the finding of the discriminative capacity of AHI in patients who have optimized treatment according to conventional guidelines identifies a new possible therapeutic target on which to act.

However, we also admit to certain limitations. First of all, this is a study with a cross-sectional design, therefore a causal relationship between OSA and renal function impairment cannot be definitively established. Second, a central laboratory has not been used for analytical determinations or sleep studies. We opted for local analyses performed at each study center to better reflect a situation of daily clinical practice, while also taking into account the fact that the use of both procedures is practically universal. Although this may detract from our results, we believe that it could increase their external validity. Third, as in most previous studies, the UACR was determined from one urine sample rather than three different samples, as prior studies have shown that a single measurement is accurate for assessing the degree of nephropathy and is therefore sufficient for clinical or epidemiological studies^42^. Fourth, our study population was exclusively Caucasian European, so its extrapolation to other ethnic groups should be done with caution.

In conclusion, the presence of severe OSA in patients with DKD is associated with a higher UACR and a lower eGFR, reflecting a greater severity of renal involvement. Furthermore, in patients with optimized treatment, AHI and oxygen desaturation index are selected as independent determinants of UACR and eGFR, which raises the possibility of identifying a new therapeutic target on which to act in these patients to prevent their progression to end-stage renal disease.

## Methods

### Study subjects

A multicenter, observational cross-sectional study was conducted in six teaching hospitals in Spain. Patients were consecutively selected according the following criteria: age between 18 and 80 years old, overweight or obese (body mass index [BMI] greater than or equal 25 kg/m^2^), previous diagnosis of type 2 diabetes (current treatment with oral anti-diabetic drugs and/or insulin, fasting glucose level > 126 mg/dl on at least two occasions, blood glucose level 2 h after oral glucose tolerance test ≥ 200 mg/dl, or glycated hemoglobin (HbA1c) > 6.5%) and diabetic kidney disease (UACR > 30 mg/g, eGFR < 60 ml/min/1.73 m^2^, or urinary albumin excretion ≥ 30 mg/24 h). Moreover, patients were on stable diabetes treatment, and angiotensin-converting enzyme (ACE) inhibitors, angiotensin II receptor blockers (ARB) or aldosterone receptor antagonists (MRA) were used at a recommended therapeutic dose for at least 4 weeks.

As exclusion criterion was considered the presence of: UACR > 3000 mg/g; severe daytime sleepiness (Epworth sleepiness scale > 18); acute coronary syndrome, stroke, transient ischemic attack, or hospitalization for heart failure worsening within the previous 30 days; type 1 diabetes or known non-diabetic renal disease; evidence of bilateral renal artery stenosis; systolic blood pressure ≥ 180 mmHg or diastolic blood pressure ≥ 110 mm Hg. Dialysis for acute renal failure within the 6 previous months, continuous treatment with non-steroidal anti-inflammatory drugs, treatment with high doses of acetylsalicylic acid (> 500 mg/day), or previous treatment with CPAP were considered additional exclusion criteria.

The study was approved by the Institutional Ethics Committee of the Hospital Universitario La Paz, Madrid, Spain (number of protocol: PI-2386), and all subjects gave their written informed consent. All methods were performed in accordance with the relevant guidelines and regulations.

### Clinical evaluation

Anthropometric characteristics were measured, including neck circumference and waist–hip ratio as well as body composition (BF511 monitor, Omron Healthcare, Kyoto, Japan). Based on self-administered questionnaires and medical records, duration of diabetes, DKD, smoking status and comorbidities were recorded. ‘Smokers’ were defined as subjects who currently smoked more than ten cigarettes per day. Past smokers who had quit smoking were not considered smokers in this study. Aged-adjusted Charlson comorbidity index was calculated^43^. All medications used by the participants at the time of the sleep test were listed.

Three consecutive heart rate and blood pressure readings were obtained with an automatic device in the seated position after the subjects had rested for at least 5 min. The Epworth sleepiness scale^44^ and the International Physical Activity Questionnaire (iPAQ)^45^ were used to assess baseline sleepiness and daily physical activity, respectively.

### Sleep study

All patients underwent overnight respiratory polygraphy with validated portable devices providing continuous recording of oronasal flow and pressure, thoracic and abdominal respiratory movements, heart rate and arterial oxygen saturation (SaO_2_)^46^. Sleep studies with < 4 h of adequate recordings were repeated and excluded if the quality remained poor. All readings and scoring were conducted manually by experienced, trained personnel. Apnea was defined as an interruption of oronasal flow of > 10 s; presence or absence of thoracic and abdominal movements made a distinction between central and obstructive events, respectively^47^. Hypopnea was defined as a 30–90% reduction in oronasal airflow for > 10 s, associated with oxygen desaturation ≥ 3%^46,47^. AHI was defined as the number of apneas plus hypopneas per hour of recording, while tSaO_2_ < 90% was defined as the percentage of recording time with SaO_2_ < 90%. In addition, mean saturation, lowest saturation and oxygen desaturation index (ODI) were measured. According to AHI, patients were classified as non-OSA (< 5/h), mild OSA (5–14.9/h), moderate OSA (15–29.9/h) or severe OSA (≥ 30/h)^48^.

### Laboratory measurements

Single early-morning urine and blood samples were obtained after the sleep study and processed at each local laboratory. Urine albumin was measured by a nephelometric assay (assay sensitivity 2 mg/l), and creatinine was measured by a kinetic colorimetric assay. Microalbuminuria was defined as UACR > 30 mg/g, and macroalbuminuria was defined as > 300 mg/g. The coefficients of reproducibility for the UACR measurement were 2% intra-assay, 6% interassay and 12% intraindividual in our laboratory. Urine samples with evidence of urinary tract infection were repeated when free from infection.

Serum creatinine was measured by photometric method, and eGFR was calculated using the Chronic Kidney Disease Epidemiology (CKD-EPI) creatinine equation: eGFR = 141 × min(S_cr_/κ, 1)^α^ × max(S_cr_/κ, 1)^−1.209^ × 0.993^Age^ × 1.018 [if female], where: S_cr_ is serum creatinine in mg/dl, κ is 0.7 for females and 0.9 for males, α is − 0.329 for females and − 0.411 for males, min indicates the minimum of S_cr_/κ or 1, and max indicates the maximum of S_cr_/κ or 1^49^. HbA1c was determined using a high-performance liquid chromatography method certified by the National Glycohemoglobin Standardization Program (NGSP). Fasting glucose and insulin levels were also measured and, in noninsulin users, Homeostasis Model Assessment-Insulin Resistance (HOMA-IR)^50^ and Qualitative Insulin Sensitivity Check Index (QUICKI) scores^51^ were calculated. Finally, total cholesterol, high-density lipoprotein cholesterol (HDL-c), low-density lipoprotein cholesterol (LDL-c), triglycerides and high sensitivity C-reactive protein (hsCRP) were determined at the different participating hospitals using standard enzymatic methods.

### Statistical analysis

Accepting an alpha risk of 0.05 and a beta risk of 0.1 in a two-sided test, the required sample size to detect a correlation coefficient between AHI and urinary albumin–creatinine ratio of at least 0.22 was 214 subjects.

Continuous variables are expressed as mean ± standard deviation or median (interquartile range [IQR]), depending on their distribution, while categorical variables are reported as absolute numbers and percentages. Normality in the distribution of the data for each variable was explored using Kolmogorov–Smirnov and Shapiro–Wills tests. Differences between groups were analyzed by the chi-square test or Fisher exact test (categorical variables), Kruskal–Wallis test (ordinary or non-normal metric variables) and ANOVA with post-hoc comparisons by the Bonferroni test (normal metric variables).

To assess relationships between variables, non-normal variables were log transformed and Spearman correlation test was used. Significant contributors to UACR or eGFR were introduced in a forward stepwise multiple linear regression analysis to identify independent determinants. In this analysis, predictor variables were retained only if their addition significantly improved (*p* < 0.05) the fraction of explained variability (r^2^). Changes in the distribution of the residuals, residual standard deviation, and the homogeneity of the variance over the predictors were also explored. All statistical tests were two-sided and statistical significance was assumed for *p* < 0.05. Statistical Package for the Social Sciences, version 26.0 software (SPSS Inc., Chicago, IL, USA) was employed.
